# Risk factors of infection after transarterial chemoembolization for hepatocellular carcinoma: A protocol for systematic review and meta-analysis

**DOI:** 10.1097/MD.0000000000025851

**Published:** 2021-05-21

**Authors:** Zhipeng Shi, Wen Yang, Hao Tang, Xiuhong Li

**Affiliations:** The People's Hospital of Dazu District Chongqing, Dazu, Chongqing, China.

**Keywords:** hepatocellular carcinoma, infection, meta-analysis, protocol, risk factors, transarterial chemoembolization

## Abstract

**Background:**

Transarterial chemoembolization (TACE) has the characteristics of minimally invasive, strong repeatability, and good curative effect, so it is commonly used in the nonoperative treatment of hepatocellular carcinoma (HCC). However, infection will occur after TACE, which not only increases the hospitalization time and medical expenses, but also affects the efficacy of TACE treatment. At present, there is a lack of analysis of the risk factors of infection after TACE of patients with HCC. In this study, meta-analysis was used to further explore the risk factors of postoperative infection in patients with HCC after TACE, and to provide strategies for infection prevention and intervention.

**Methods:**

To search the literatures about the influencing factors of post-TACE infection in patients with HCC published from the establishment of PubMed, Embase, The Cochrane Library, Web of Science, China Biology Medicine Database, China National Knowledge Infrastructure, China Science and Technology Journal Database, and WANFANG to April 2021. Screening was carried out according to inclusion criteria and exclusion criteria. A meta-analysis was performed using RevMan 5.3 software.

**Results:**

We disseminated the findings of this systematic review and meta-analysis via publications in peer-reviewed journals.

**Conclusion:**

This study systematically reviewed the existing evidence and determined the incidence and predictors of infection after TACE of patients with HCC.

**Ethics and dissemination:**

The private information from individuals will not be published. This systematic review also should not damage participants’ rights. Approval from an ethics committee is not required for this study. The results may be published in a peer-reviewed journal or disseminated in relevant conferences.

**OSF Registration number:**

DOI 10.17605/OSF.IO/26P5X

## 1 Introduction

Hepatocellular carcinoma (HCC) is the fifth largest malignant tumor in the world, and its incidence is increasing every year.^[1]^ It is estimated that there will be more than 1 million new cases of HCC every year by the year of 2025, bringing a huge health burden to people all over the world.^[2]^ China is a large country for HCC,^[3]^ with 55% of the world's HCC patients in China. HCC has become the second leading cause of cancer death among Chinese residents.^[4]^ Currently, a variety of approaches and disciplines coexist in the treatment of HCC using liver transplantation, surgical resection, minimally invasive interventions, and drug combination therapy.^[5]^ As most of the patients are complicated with liver cirrhosis, or the symptoms are in the middle and late stage, the chance of surgical resection is only 20% to 30%.^[6]^ Transarterial chemoembolization (TACE) has received increasing attention because of its minimally invasive, effective, and reproducible features.^[7]^

Most of the patients treated with TACE are in the middle and advanced stage of HCC, of which immune function is low, coupled with the damage to local tissue caused by invasive operation and the inhibition of immune function caused by chemotherapeutic drugs, leading to postoperative infection of TACE and seriously affect the prognosis of patients with HCC.^[8]^ Therefore, to explore the influencing factors of postoperative infection of HCC after TACE has become one of the problems that must be solved in the treatment of HCC patients with TACE.

Identifying the risk factors of infection after TACE is helpful to accurate infection prevention and controlling and solving the problem. Domestic and foreign reports on the prevention and treatment of infection after TACE are inconsistent, and so are the mentioned risk factors of infection.^[9–14]^ In this study, to screen out the risk factors of infection and provide a reference basis for clinical prevention and reduction of post-TACE infection in patients with HCC, meta-analysis was conducted on the published literature on the risk factors of post-TACE infection in patients with HCC.

## 2 Methods

### 2.1 Study registration

This protocol has been registered on Open Science Framework grant number (OSF Registration number: DOI 10.17605/OSF.IO/26P5X). This report is based on the preferred reporting items for systematic review and meta-analysis protocols.^[15]^

### 2.2 Eligibility criteria

#### 2.2.1 Inclusion criteria

1. Participants: patients with HCC diagnosed by pathology and in accordance with the indications of TACE and without indications for hepatectomy; those with acute or chronic infection or with severe ascites, hepatic encephalopathy and other malignant tumors before TACE treatment were excluded, and previous partial hepatectomy was excluded.
2. Research content: study on risk factors of infection after TACE of patients with HCC.
3. Type of study: case-control or cohort study.
4. Outcome indicators: the risk factors were reported in the literature with original data, and the specific values of relative risk and 95% confidence interval of risk factors could be extracted.

#### 2.2.2 Exclusion criteria

1. Literatures that are repeatedly published or quote the same unit of data;
2. Literatures without original data or incomplete data records;
3. Reviews, basic research, dissertations, conference papers;
4. Literatures that cannot be obtained full text.

### 2.3 Search strategy

Conventional literature retrieval strategies were used to retrieve the literatures on risk factors analysis of HCC infection after TACE from PubMed, Embase, Cochrane Library, Web of Science, China Biology Medicine Database, China National Knowledge Infrastructure, China Science and Technology Journal Database, and WANFANG. The retrieval time is from the establishment to April 2021. These search terms are summarized in Table 1.

**Table 1:** Search strategy used in PubMed database.

Number	Search terms
#1	Carcinoma, hepatocellular [MeSH]
#2	Hepatocellular carcinoma [Title/Abstract]
#3	Hepatoma [Title/Abstract]
#4	Liver cancer, adult [Title/Abstract]
#5	Liver cell carcinoma [Title/Abstract]
#6	Liver cell carcinoma, adult [Title/Abstract]
#7	Adult liver cancer [Title/Abstract]
#8	Adult liver cancers [Title/Abstract]
#9	Cancer, adult liver [Title/Abstract]
#10	Cancers, adult liver [Title/Abstract]
#11	Carcinoma, liver cell [Title/Abstract]
#12	Carcinomas, hepatocellular [Title/Abstract]
#13	Carcinomas, liver cell [Title/Abstract]
#14	Cell carcinoma, Liver [Title/Abstract]
#15	Cell carcinomas, liver [Title/Abstract]
#16	Hepatocellular carcinomas [Title/Abstract]
#17	Hepatomas [Title/Abstract]
#18	Liver cancers, adult [Title/Abstract]
#19	Liver cell carcinomas [Title/Abstract]
#20	or/1–19
#21	Transarterial chemoembolization [Title/Abstract]
#22	TACE[Title/Abstract]
#23	Hepatic arterial chemoembolization [Title/Abstract]
#24	Transcatheter hepatic arterial chemoembolization [Title/Abstract]
#25	or/21–24
#26	Infection [Title/Abstract]
#27	Infections [Title/Abstract]
#28	or/26–27
#29	Risk factor [Title/Abstract]
#30	Risk assessment [Title/Abstract]
#31	Multivariate analysis [Title/Abstract]
#32	Multivariable logistic regression [Title/Abstract]
#33	or/29–32
#34	#20 and #25 and #28 and #33

### 2.4 Study selection

**Figure 1:**
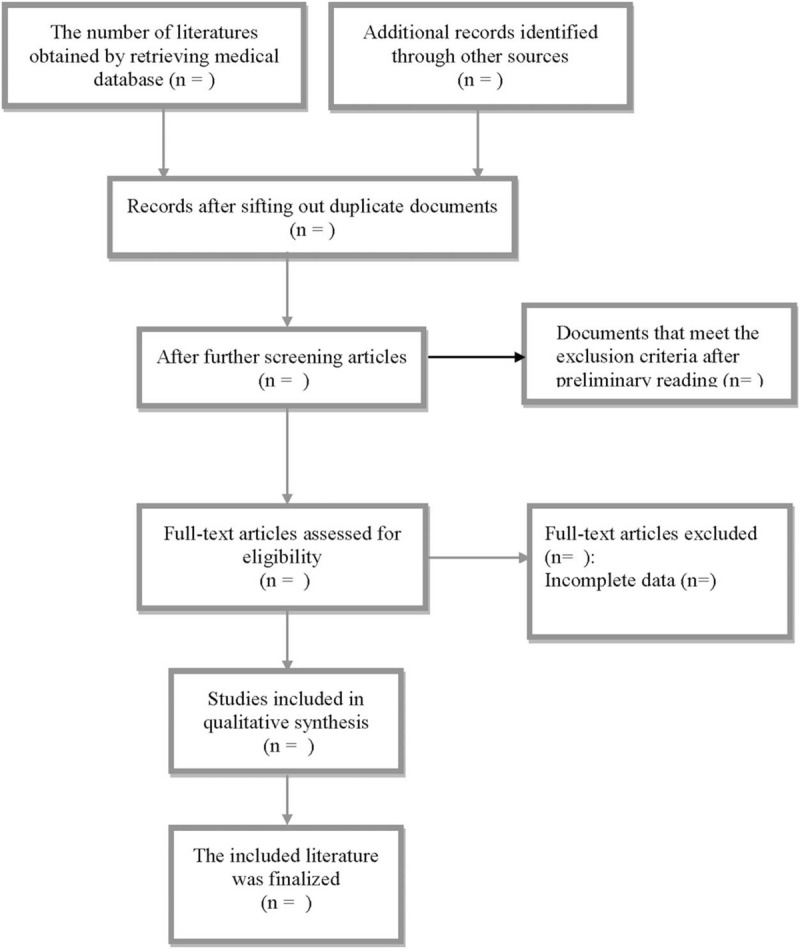
Flow chart of study selection.

Two researchers independently screened and extracted data according to the inclusion and exclusion criteria of the literature. In case of disagreement, the 2 parties have discussed and resolved the matter or sought the opinions of a third party. The process of the selection is exhibited in Figure 1.

### 2.5 Data extraction

Data extraction includes: publication time, first author, type of study, sex, age, sample size, related risk factors, and so on.

### 2.6 Assessment of the risk of bias

Two researchers independently used the Newcastle–Ottawa scale^[16]^ to evaluate the included study. The full score of 9 stars and ≥7 stars indicates that the quality of the literature is high.^[17]^ After the evaluation, the 2 researchers discussed it. In case of disagreement, they have discussed and decided with the third researcher.

### 2.7 Data analysis

Meta-analysis was performed using RevMan 5.3 software. All the variables included in the study were binary variables, expressed by relative risk and 95% confidence interval. If there are no findings of statistical heterogeneity, the Mantel–Haenszel fixed effect model would be adopted for data synthesis.^[18]^ If there is significant statistical heterogeneity, we would apply the DerSimonian–Laird random effect model.^[19]^

### 2.8 Assessment of heterogeneity

The magnitude of heterogeneity in the results was determined by *χ2* test and *I*^*2*^ quantitative analysis. When *P* < .1, and (or) *I*^*2*^ > 50%, the random effect model would be adopted for the combined analysis. Otherwise, the fixed effect model would be adopted.

### 2.9 Subgroup analysis

Subgroup analysis was conducted according to race, literature quality, and sample size.

### 2.10 Sensitivity analysis

The stability of the results was tested by removing the maximum weight from the study and looking at the change in the amount of effect.

### 2.11 Assessment of reporting biases

If no less than 10 articles are included, we would use funnel chart to analyze the publication bias.^[20]^

### 2.12 Management of missing data

The object of the study is the defects of the original data. We contacted the author by email and asked for the original data. If the original data was not available, we analyze the existing data.

### 2.13 Ethical review and informed consent of patients

The content of this article do not involve moral approval or ethical review and will be presented in print or at relevant conferences.

## 3 Discussion

TACE therapy has become a recognized nonoperative therapy at home and abroad.^[21,22]^ The injection of chemotherapy drugs and embolic drugs into the hepatic artery of HCC patients through a catheter is conducive to increasing the local concentration of drugs, effectively blocking the blood supply, and killing cancer cells, thereby improving the survival quality of patients with advanced HCC who have missed the chance of surgery.^[23–26]^ Puncture, intubation, and drug injection should be performed during TACE, which is easy to cause certain trauma to the respiratory tract, digestive tract, skin, and soft tissue of the patients. In addition, chemotherapeutic drugs can lead to side effects such as nausea, vomiting, and myelosuppression, which can reduce the body resistance and cause infection.

According to the Shawker study, the incidence of transient fine bacterial infection associated with TACE was 4%, while that of liver abscess was 1.4%.^[27]^ The death rate of liver abscess after TACE was 13.4% to 50.0% without proper treatment.^[9,28–30]^ In clinic, the ratio of application of prophylactic antibiotics in TACE intervention is confused, and the types and time of application are different. Therefore, it is important to explore the risk factors of infectious complications after TACE for HCC and to find ways to reduce or avoid infectious complications after TACE. In this study, meta-analysis was used to analyze the risk factors of post-TACE infection in patients with HCC, which provided a basis for the prevention and treatment of post-TACE infection.

## Author contributions

**Data collection:** Zhipeng Shi.

**Funding acquisition:** Xiuhong Li.

**Investigation:** Hao Tang.

**Resources:** Wen Yang and Hao Tang.

**Software:** Zhipeng Shi.

**Supervision:** Hao Tang, Xiuhong Li.

**Writing – original draft:** Zhipeng Shi and Xiuhong Li.

**Writing – review & editing:** Wen Yang and Hao Tang.
